# Rapid detection of pneumothorax by ultrasonography in patients with multiple trauma

**DOI:** 10.1186/cc5004

**Published:** 2006-08-01

**Authors:** Mao Zhang, Zhi-Hai Liu, Jian-Xin Yang, Jian-Xin Gan, Shao-Wen Xu, Xiang-Dong You, Guan-Yu Jiang

**Affiliations:** 1 Department of Emergency Medicine, Second Affiliated Hospital, Zhejiang University, School of Medicine and Research Institute of Emergency Medicine, Zhejiang University, Hangzhou, China; 2 Department of Ultrasound, Second Affiliated Hospital, Zhejiang University, School of Medicine, Hangzhou, China

## Abstract

**Introduction:**

Early detection of pneumothorax in multiple trauma patients is critically important. It can be argued that the efficacy of ultrasonography (US) for detection of pneumothorax is enhanced if it is performed and interpreted directly by the clinician in charge of the patients. The aim of this study was to assess the ability of emergency department clinicians to perform bedside US to detect and assess the size of the pneumothorax in patients with multiple trauma.

**Methods:**

Over a 14 month period, patients with multiple trauma treated in the emergency department were enrolled in this prospective study. Bedside US was performed by emergency department clinicians in charge of the patients. Portable supine chest radiography (CXR) and computed tomography (CT) were obtained within an interval of three hours. Using CT and chest drain as the gold standard, the diagnostic efficacy of US and CXR for the detection of pneumothorax, defined as rapidity and accuracy (sensitivity, specificity, positive predictive value, negative predictive value), were compared. The size of the pneumothorax (small, medium and large) determined by US was also compared to that determined by CT.

**Results:**

Of 135 patients (injury severity score = 29.1 ± 12.4) included in the study, 83 received mechanical ventilation. The time needed for diagnosis of pneumothorax was significantly shorter with US compared to CXR (2.3 ± 2.9 versus 19.9 ± 10.3 minutes, *p *< 0.001). CT and chest drain confirmed 29 cases of pneumothorax (21.5%). The diagnostic sensitivity, specificity, positive and negative predictive values and accuracy for US and radiography were 86.2% versus 27.6% (*p *< 0.001), 97.2% versus 100% (not significant), 89.3% versus 100% (not significant), 96.3% versus 83.5% (*p *= 0.002), and 94.8% versus 84.4% (*p *= 0.005), respectively. US was highly consistent with CT in determining the size of pneumothorax (*Kappa *= 0.669, *p *< 0.001).

**Conclusion:**

Bedside clinician-performed US provides a reliable tool and has the advantages of being simple and rapid and having higher sensitivity and accuracy compared to chest radiography for the detection of pneumothorax in patients with multiple trauma.

## Introduction

Pneumothorax is a common finding in the trauma setting and affects more than 20% of major blunt trauma victims [1]. Tension pneumothorax is a serious situation that can potentially lead to cardiac arrest, requiring early diagnosis and urgent treatment. A small or medium pneumothorax is generally not life threatening, but delays in diagnosis and treatment may result in progression of respiratory and circulatory compromise in unstable patients with multiple trauma. Therefore, early detection of pneumothorax in severely injured patients, especially those who are mechanically ventilated, is of critical clinical importance.

Portable chest radiography (CXR) has been demonstrated to be an insensitive examination for the detection of pneumothorax that can miss over half of all post-traumatic pneumothorax [2,3]. Computed tomography (CT) is considered as the gold standard for the detection of pneumothorax. However, it requires severely injured patients to be transported the CT room, and is usually time-consuming, resulting in delayed diagnosis. Ultrasonography (US) can be easily performed at the bedside. With the advancement of technology, ultrasound devices have decreased in size, weight and cost, and have increased in image quality. US offers the possibility for clinicians to perform rapid evaluation of severely injured patients. The use of it to detect pneumothorax has been shown to have a higher sensitivity and specificity compared to CXR [4-6].

In multiple trauma patients, it can be argued that the efficacy of US for detection of pneumothorax is enhanced if it is performed and interpreted directly by the clinician in charge, who is familiar with the patient's condition. Reducing the time taken for bedside diagnosis of pneumothorax could allow the clinician to take earlier treatment measures. However, the ability of emergency department clinicians to perform lung US has never been evaluated and the time needed for bedside US, CXR and CT have not been compared.

We conducted the present study to assess the ability of appropriately trained emergency department clinicians to perform bedside US to rapidly detect and assess the size of pneumothorax in patients with multiple trauma. US was compared to bedside CXR and chest CT scanning.

## Materials and methods

### Study design

This is a prospective study conducted over a 14 month period from September 2004 to October 2005. The study protocol was approved by the Ethical Committee of the hospital, where informed consent was not necessary as results from the clinician-performed US alone would not have changed the therapy. Patients with multiple trauma in either the resuscitation room or the emergency intensive care unit (EICU) were enrolled. Those with subcutaneous emphysema and/or cardiac arrest following probable tension pneumothorax were excluded from the study.

In this hospital, multiple trauma patients receive initial assessment and treatment in the resuscitation room, and are then admitted to the EICU. Emergency department clinicians are directly in charge of the patients and are rotated from the resuscitation room to the EICU regularly. For patients in the resuscitation room, US was performed after initial rapid assessment by physical examination and essential resuscitation. US was conducted in all patients admitted to the EICU and in hospitalized patients with impairment of lung function requiring a chest CT scan. Three emergency department clinicians (authors MZ, ZHL and JXY) who performed bedside US had received formal training on emergency bedside US. This training comprised a 28 hour course developed by our institute based on the US emergency medicine guidelines issued by the American College of Emergency Physicians in 2001 [7]. Before performing the US, these clinicians were unaware of radiographic and CT findings.

Portable CXR (AD125P-MUXH, Shimadzu Co., Kyoto, Japan) and CT scans were performed before or after US, with an interval of less than three hours. Both were obtained with patients in the supine position. Chest CT was acquired with a 16-slice spiral CT scanning unit (Volume Zoom, Siemens Co., Forchheim, Germany). The results of chest CT and radiography were interpreted by independent radiologists who were unaware of patients' conditions and the findings of US.

In patients with clinical suspicion of large or tension pneumothorax requiring immediate chest tube placement, and in whom the clinical situation precluded performing a CT scan, the chest tube was placed after US and/or CXR. Pneumothorax was then confirmed by air bubbles released from the chest tube. In these patients, chest drain was considered the golden standard and analyzed together with the CT scan.

### Diagnosis of pneumothorax by lung ultrasonography

**Figure 1:**
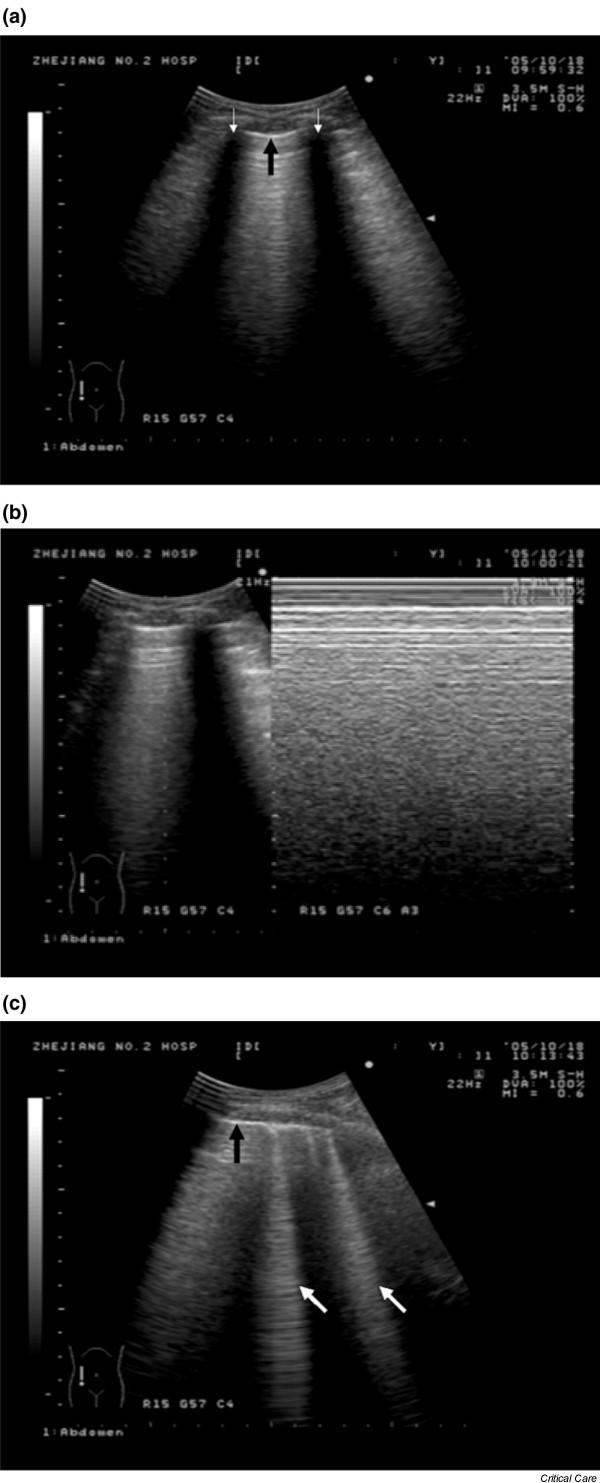
Conventional ultrasonic signs in the lung. **(a) **The pleural line (black bold arrow) is a roughly horizontal hyper-echoic line between upper and lower ribs, identified by acoustic shadows (white arrow). **(b) **Lung-sliding is a forward-and-back movement of visceral pleura against parietal pleura in real-time motion. In time-motion mode, it includes motionless parietal tissues over the pleural line and a homogenous granular pattern below it (right image). **(c) **Comet-tail artifacts (white bold arrows) are hyper-echoic reverberation artifacts arising from the pleural line, laser-beam-like and spreading up to the edge of the screen.

A portable ultrasound device (SSD-900, Aloka Co., Tokyo, Japan) is regularly used in our department, and is available at any moment. A 3.5 MHz convex probe and occasionally a 7.5 MHz linear one were used. Patients were kept in a supine position and an examination of the anterior, lateral and posterior thoraces was performed. Bilateral ultrasonic images were compared and the following characteristic signs were identified in either real-time or time-movement mode (Figure 1).

#### Pleural line

When the transducer was placed across the ribs longitudinally, the location of the ribs allowed for the accurate delineation of the pleural line, a roughly horizontal hyper-echoic line between the upper and lower ribs. Even visceral pleura and parietal pleura could be distinguished clearly with a higher frequency probe.

#### Lung sliding

A forward-and-back movement of visceral pleura against parietal pleura, caused by the respiratory excursion of the lung toward the abdomen, was detected. It was unique in the time-motion mode, characterized by a 'seashore sign', which included motionless parietal tissue over the pleural line and a homogenous granular pattern below it [8].

#### Comet-tail artifacts

A hyper-echoic reverberation artifact arose from the pleural line, laser-beam-like and well defined, spreading up to the edge of the screen. The presence of comet-tail artifacts usually indicates alveolar and/or interstitial pulmonary edema [9]. Pneumothorax was considered when the absence of both lung-sliding and comet-tail artifact was noted.

The size of pneumothorax was determined and classified as small (<30%), medium (30% to 70%) and large (>70%). For lung CT, it was determined by the ratio between the volume of pneumothorax and that of the pleural cavity, which could be automatically measured by delineating the edge of the pneumothorax and pleural cavity at different CT slices on the CT workstation. For lung US, the size of pneumothorax was determined as follows: the normal pleuro-pulmonary interface or the edge of the pneumothorax lies in the anterior, lateral or posterior chest, depending on the extension of the pneumothorax. At that point, normal lung-sliding and pneumothorax coexisted in a single view, forming 'partial lung-sliding' [10]. This phenomenon was described as 'lung point' [11], where lung-sliding and absent lung-sliding appeared alternately. The size of pneumothorax was inferred by ascertaining such points at different intercostal spaces. When these points are lined up, the contour of the pneumothorax is also outlined.

### Statistical analysis

Data were expressed as mean ± standard deviation and analyzed by statistical software SPSS13.0 (SPSS Inc., Chicago, IL, USA). The performance of US and CXR for the detection of pneumothorax was compared to the gold standard (CT + chest drain) using a Kappa agreement test. A *Kappa *value less than 0.40 indicates low agreement, while a value greater than 0.75 indicates close agreement with the gold standard [12]. The duration for acquisition of US and CXR were compared with a paired Student *t* test. A *p *value less than 0.05 was considered as statistically significant.

Sensitivity = true positive/(true positive + false negative); specificity = true negative/(true negative + false positive); positive predictive value = true positive/(true positive + false positive); negative predictive value = true negative/(true negative + false negative); false positive ratio = false positive/(true negative + false positive); false negative ratio = false negative/(true positive + false negative); diagnostic accuracy = (true positive + true negative)/(true positive + true negative + false positive + false negative). The diagnostic sensitivity, specificity, positive predictive value, negative predictive value and accuracy for US and CXR were calculated and then compared by Chi-square test or Fisher's exact test.

## Results

### Patients

Ultrasonography was performed in 163 patients with multiple trauma. Of these, 28 were excluded for an absence of chest CT or because the interval between US and CT scan was more than three hours. Of 135 patients included, 31 were in the resuscitation room and 104 in the EICU; 114 were male and 21 were female. The average age was 45 ± 15 years. All patients suffered from blunt trauma, including traffic accident (61.5%), falls (20.7%), crush injuries (9.6%) and others (8.2%). There were 83 patients (61.5%) who received mechanical ventilation. The average injury severity score was 29.1 ± 12.4 (range 16 to 41), and the average acute physiology and chronic health evaluation (APACHE) II score at admission was 19.9 ± 11.6 (range 9 to 36).

### Performance of ultrasonography and radiography compared to the gold standard (CT scan and chest drain)

According to the gold standard (131 patients with CT and four patients with chest drain), pneumothorax was present in 29 of the 135 trauma patients (21.5%), of which three had bilateral pneumothorax. Pneumothorax was diagnosed by US in 28 patients as the absence of both lung-sliding (*n *= 31) and comet-tail artifacts (*n *= 43), two of them presenting with bilateral pneumothoraces. The sensitivity, negative predictive value and diagnostic accuracy of US were significantly higher compared to CXR (Table 1). Kappa agreement test indicated US had a stronger agreement with CT (*Kappa *= 0.844, *p *< 0.001) compared to CXR (*Kappa *= 0.374, *p *< 0.001).

**Figure 2:**
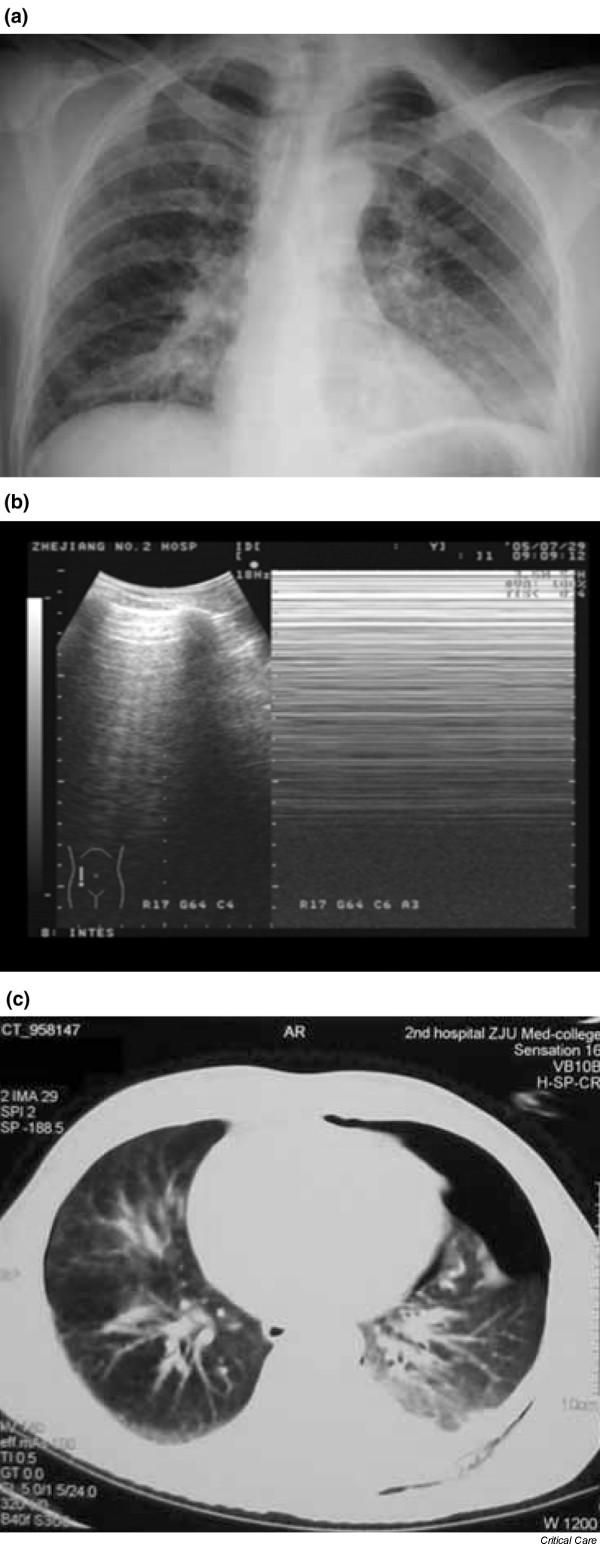
A typical patient with pneumothorax correctly diagnosed by US and missed by CXR. This 42 year old male patient sustained injuries from a car accident, and arrived with dyspnea, tachycardia, hypotension and desaturation requiring mechanical ventilation. **(a) **The supine chest radiograph did not enable a diagnosis of pneumothorax. **(b) **A rapid exploration of the thorax by US indicated medium left pneumothorax (absence of lung-sliding), associated with left lung contusion and pleural effusion. **(c) **The diagnosis was confirmed afterwards by chest CT. Arterial oxygenation was improved after chest tube placement.

In 21 true positive patients diagnosed by US and confirmed by CT, 11 patients had small, 7 had medium and 3 had large pneumothoraces, in close agreement with the results from CT (*Kappa *= 0.669, *p *< 0.001; Table 2). In three false positive patients, one developed severe late acute respiratory distress syndrome and two had adhesion of pleura. False negative results were due to a small pneumothorax in three patients, and a locally separated pneumothorax in one case. CXR diagnosed pneumothorax in eight patients with medium or large pneumothorax. Among 21 false negative patients diagnosed by CXR, 19 sustained small and two had medium pneumothoraces. Figure 2 shows a typical pneumothorax correctly diagnosed by US and missed by CXR.

### Time taken for diagnosis of pneumothorax

The portable ultrasound device was readily available and the average time for US examination was 2.3 ± 2.9 minutes (range 1.5 to 7 minutes). The time interval between requesting a CXR and obtaining access to it was 12.4 ± 6.7 minutes (range 5 to 23 minutes), and another 7.5 ± 3.8 minutes (range 6 to 11 minutes) were needed to get the results. US allowed a significantly quicker detection of pneumothorax compared to CXR (2.3 ± 2.9 minutes versus 19.9 ± 10.3 minutes, *p *< 0.001). In 43 patients in whom the time needed for CT scan was recorded, the duration (transportation plus CT scanning plus oral report) was significantly longer than that for US (16.3 ± 7.8 minutes versus 2.5 ± 2.8 minutes, *p *< 0.001). If the interval between requesting the CT scan and transportation of patients was taken into account, this time would be even longer.

### Clinical outcome and management of pneumothorax

In 29 patients with pneumothorax, 21 presented with at least one chest injury, including hemothorax, lung contusion, rib fracture and contusion of the chest wall, and manifested different symptoms/signs, including dyspnea, chest pain, hypoxia and tachycardia. Four patients underwent chest tube placements for high clinical suspicion of large or tension pneumothorax; US correctly detected all four of these cases. In nine patients with large or medium pneumothorax, chest drains were placed immediately after the CT scan, allowing an improvement of symptoms and oxygenation in seven patients. In 16 patients with small pneumothorax, chest tubes were later placed in five mechanical ventilated patients owing to progression of the pneumothorax.

## Discussion

The present study demonstrates that, in multiple trauma patients, bedside lung US performed by emergency department clinicians enables a rapid and reliable detection of pneumothorax compared to CXR, in particular when small and medium pneumothoraces are involved.

### Emergency department clinician-performed ultrasonography for diagnosis of pneumothorax

Ultrasound was first used to diagnose pneumothorax in humans in 1987 [13]. It was based on the principle that, without previous pleural disease, the visceral pleura moves against the parietal one during normal spontaneous breathing or mechanical ventilation. This physiological movement can be detected by ultrasound, forming lung-sliding in real-time and time-motion modes [14]. Comet-tail artifacts are vertical reverberation artifacts arising from the visceral pleura, and caused by swollen septa surrounded by air. It is usually thought to be a pathological sign, and multiple comet-tail artifacts in one view can indicate alveolar or interstitial syndrome [9]. When pneumothorax is present, the pleura is separated by air, which hampers the transmission of the ultrasound beam, so neither lung-sliding nor comet-tail artifacts can be observed. It has been demonstrated that the absence of lung-sliding alone has a high sensitivity, specificity, negative predictive value and positive predictive value for the detection of pneuomothorax [14]; the absence of comet-tail artifacts alone has a sensitivity and negative predictive value up to 100% [15]. A higher diagnostic accuracy was obtained when both lung sliding and comet-tail artifacts were absent [15].

Pneumothorax occurs commonly in trauma patients. It mainly results from direct chest trauma, barotrauma following mechanical ventilation and invasive procedures. Because the emergency department clinician in charge is familiar with the patient's condition, it can be argued that the efficacy of US is enhanced if it is performed and interpreted directly by the clinician [16,17]. Recent practice management recommended that US be considered as the initial modality to exclude hemoperitoneum [18]. Consequently, Kirkpatrick and colleagues [19] suggested that examining the chest to diagnose pneumothorax should be a natural progression of this acceptance of trauma sonography performed by clinicians.

In the present study, US rapidly detected 25 of 29 patients presenting pneumothorax while CXR diagnosed only 8 cases. Our results from a large series of trauma patients confirm previous studies [4-6,19] and demonstrate that bedside US performed by the clinician in charge provides a higher sensitivity and accuracy in detection of pneumothorax than portable supine CXR. Another clinically relevant finding is that lung US enables a significantly quicker diagnosis of pneumothorax compared to portable CXR and CT. Emergency bedside CXR is available to all wards in our hospital, but its access is often delayed owing to increased calls and limited staff numbers, especially in the evening. Because clinician-performed US had only been newly introduced to our department, clinical decisions were made only after CXR and/or CT were performed. Although no obvious adverse outcomes were related to the delay this caused, we felt that the decreased time required for US could allow clinicians to take earlier medical measures in the treatment of pneumothorax and other traumatized organs. This was deemed to be particularly beneficial in unstable patients.

It should be pointed out that the time reported for performing US was solely for detection of pneumothorax. The time for a thorough exploration of the entire lung, including pleural effusion and lung consolidations, needs further evaluation.

### Accuracy of detection of pneumothorax by lung ultrasonography

In this study, a sensitivity of 86% and a specificity of 97% were obtained when using US for the detection of pneumothorax. Three patients had false positive and four patients had negative diagnoses of pneumothorax. Some factors could affect the diagnostic accuracy. First, a comet-tail artifact is usually thought to be a pathological sign [9] and is absent when there are no obvious lung parenchyma injuries. Second, lung-sliding may disappear if there is previous pleural disease, which results in adhesion of visceral and parietal pleura. Kirkpatrick and colleagues [19] reported that two false positive diagnoses of left sided pneumothoraces in trauma patients with left lung atelectasis resulted from right main-stem endotracheal intubation. In our study, two of three false positive diagnoses were due to the adhesion of pleura, which was confirmed by CT. Third, a pneumothorax can be missed when its size is small or it is a locally separated one. When the patient is in a supine position, a small pneumothorax usually locates in the antero-apical or antero-basal space [20]. As a result, examination limited to the second intercostal space is not sufficient for a diagnosis. In our study, exploration of the entire thorax was performed; however, we still missed the diagnosis of three small pneumothoraces. Another explanation is that chest muscle contraction during spontaneous breathing could render lung-sliding difficult to interpret. Temporary paralysis of mechanically ventilated patients could help to ascertain the diagnosis. A higher frequency probe was thought to be superior for the detection of small pneumothoraces [6]. We mainly used a 3.5 MHz probe in this study, and both probes were used in only 12 patients; thus, we could not compare their performance. Finally, inter- and intra-operator's variability of diagnosis could influence the results. In the present study, this variability was not tested. Further study is required to verify inter- and intra-operator's variability in patients with small and medium pneumothoraces.

### Determination of the size of pneumothorax and its clinical significance

Determination of the size of a pneumothorax is another important issue for clinical decisions in the management of pneumothorax. Classically, pneumothorax is classified as small, medium or large according to CXR or CT. Our results show that bedside supine CXR detects small pneumothoraces with an extremely low sensitivity. CT is no doubt the best technique for detection of occult pneumothorax [21,22]. However, even in hospitals with CT facilities close to the ICU, the transport of unstable trauma patients to the CT room still poses potential risks and is time-consuming.

US was initially considered as being unable to make this classification [10]. Subsequent studies have overturned this viewpoint [8,23,24]. A recent study has shown that the localization of 'lung point', where lung-sliding and absent lung-sliding appear alternately, allows the determination of the size of pneumothorax with a sensitivity of 79% [8]. Using this method, we found a good agreement between US and CT scans in determining the size of pneumothorax.

Since 1990s, the increased use of CT has resulted in at least twice the incidence of small pneumothoraces being diagnosed [25]. Our results indicate that US has a high concordance with CT in the detection of small and medium pneumothoraces. However, to date, there is still little evidence regarding how patients with small and medium pneumothoraces should be clinically managed [26]. In our study, chest tube was placed in 13 patients with medium to large pneumothoraces. Of 16 patients with small pneumothoraces, the size of the pneumothorax increased in five mechanically ventilated patients (31%), requiring subsequent chest tube placement. This result suggests that clinical early detection of occult pneumothorax allows a close follow-up of the high-risk patients.

## Conclusion

Clinician-performed US is a reliable tool for the diagnosis of pneumothorax and determination of its size in patients with multiple trauma. It holds the advantage of portability, simplicity, rapidity, and higher sensitivity and accuracy compared to CXR. US provides a useful adjunct for emergency department clinicians in treating multiple trauma patients.

## Key messages

• Clinician-performed US had higher accuracy in diagnosing pneumothorax and determining its size.

• Clinician-performed US markedly shortened the time to diagnose pneumothorax in multiple trauma patients.

• US provides a useful adjunct for emergency department clinicians in treating multiple trauma patients.

## Abbreviations

CT = computed tomography; CXR = chest radiography; EICU = emergency intensive care unit; US = ultrasonography.

## Competing interests

The authors declare that they have no competing interests.

## Authors' contributions

MZ and GYJ contributed to the study design. MZ, ZHL and JXY recruited patients, performed US and collected data. JXG and SWX arranged chest radiographs and transported patients for CT scans. MZ managed the data and drafted the manuscript. XDY provided technical support on US and checked the results.

## Figures and Tables

**Table 1:** Efficacy for diagnosing pneumothorax in multiple trauma patients by clinician-performed ultrasonography and radiography

Parameters	Ultrasonography (%)		Radiography (%)		Comparison
	Value	95%CI	Value	95%CI	P

Sensitivity	86.2 (25/29)	73.7–98.8	27.6 (8/29)	11.3–43.9	<0.001
Specificity	97.2 (103/106)	94.0–100	100 (106/106)	100–100	0.246^a^
Positive predictive value	89.3 (25/28)	77.8–100	100 (8/8)	100–100	1.0^a^
Negative predictive value	96.3 (103/107)	92.7–99.9	83.5 (106/127)	77.0–89.9	0.002
False positive ratio	2.8 (3/106)	0–6.0	0 (0/106)	0–0	0.246^a^
False negative ratio	13.8 (4/29)	1.2–26.3	72.4 (21/29)	56.1–88.7	<0.001
Accuracy	94.8 (128/135)	91.1–98.6	84.4 (114/135)	78.3–90.6	0.005

^a^Fisher's exact test. CI, confidence interval.

**Table 2:** Concordance in size determination of pneumothorax between ultrasonography and computed tomography in 21 true positive patients

	US			Total
Chest CT	Large	Moderate	Mild	(CT)

Large	2	0	0	2
Moderate	1	5	1	7
Mild	0	2	10	12
Total (US)	3	7	11	21

Kappa agreement test: *Kappa *= 0.669, *p *< 0.001. CT, computed tomography; US, ultrasonography.
